# Leprosy with Atypical Skin Lesions Masquerading as Relapsing Polychondritis

**DOI:** 10.1155/2016/7802423

**Published:** 2016-12-26

**Authors:** Punit Pruthi, Hariharan Munganda, Amit Bangia, Uma Rani, Rajesh Budhiraja, Swapnil Brajpuriya

**Affiliations:** ^1^Department of Internal Medicine and Rheumatology, Asian Institute of Medical Sciences, Faridabad, India; ^2^Department of Dermatology, Asian Institute of Medical Sciences, Faridabad, India; ^3^Department of Laboratory Services, Asian Institute of Medical Sciences, Faridabad, India; ^4^Department of ENT, Asian Institute of Medical Sciences, Faridabad, India

## Abstract

Leprosy can present with a variety of clinical manifestations depending on the immune status of the individual. After dermatological and neurological involvement, rheumatic features specially various forms of arthritis are the third most common manifestation of the disease. We describe a unique case of a 22-year-old patient presenting with external ear involvement mimicking relapsing polychondritis along with inflammatory joint symptoms and skin lesions. Ear involvement in relapsing polychondritis characteristically is painful and spares the noncartilaginous ear lobules, in contrast to painless ear involvement in leprosy affecting the lobules as well. Histopathology confirmed the diagnosis, although the ear and skin lesions were not classical of leprosy. Such a presentation of leprosy closely mimicking relapsing polychondritis has not been described previously. Tissue diagnosis should always be attempted whenever possible in patients presenting with autoimmune features, so that inappropriate therapy with immunosuppressants is avoided.

## 1. Introduction

Leprosy (Hansen's disease) is a chronic infectious disease caused by* Mycobacterium leprae*. It is still endemic in many developing countries, with majority of cases detected in countries like India, Brazil, Madagascar, and Nepal [1, 2]. It primarily affects the cooler areas of the body like peripheral nerves, skin, upper respiratory tract, external ears, and nasal mucosa. Patients suffering from leprosy exhibit a wide spectrum of presentation ranging from tuberculoid to the lepromatous pole, with the immunologically unstable borderline forms in-between, depending upon the immune status of the individual [2, 3].

The cardinal signs of leprosy include hypoaesthesia, skin lesions, and thickened peripheral nerves [3]. Rheumatological manifestations are seen in 1% to 70% of patients, resulting in clinical presentations of acute inflammatory arthritis, chronic symmetrical polyarthritis mimicking rheumatoid, Charcot's arthropathy, swollen hands and feet syndrome, tenosynovitis, vasculitis, lupus-like illness, and so forth [4, 5]. In addition various autoantibodies like antinuclear antibodies (ANA) and rheumatoid factor (RF) may be positive in leprosy, increasing the chances of misdiagnosis as a true autoimmune disease resulting in inappropriate treatment with immunosuppressive therapy [5].

In this report we describe a young male presenting with features of relapsing polychondritis (RPC), a rare autoimmune disease.

## 2. Case Presentation

**Figure 1:**
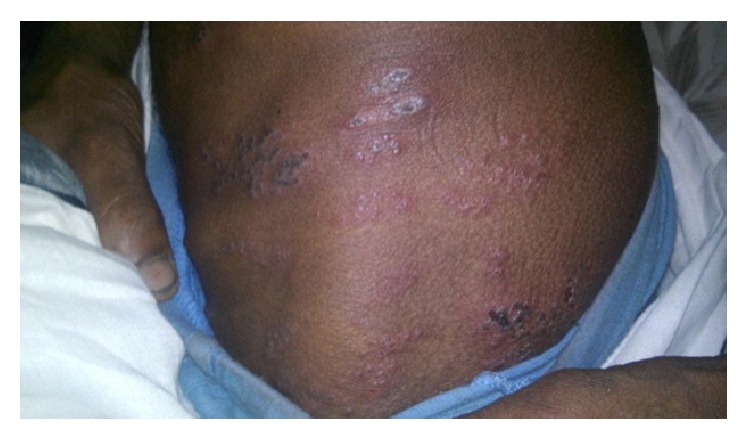
Erythematous, scaly, and ulcerative rash with irregular margins present over elbow.

**Figure 2:**
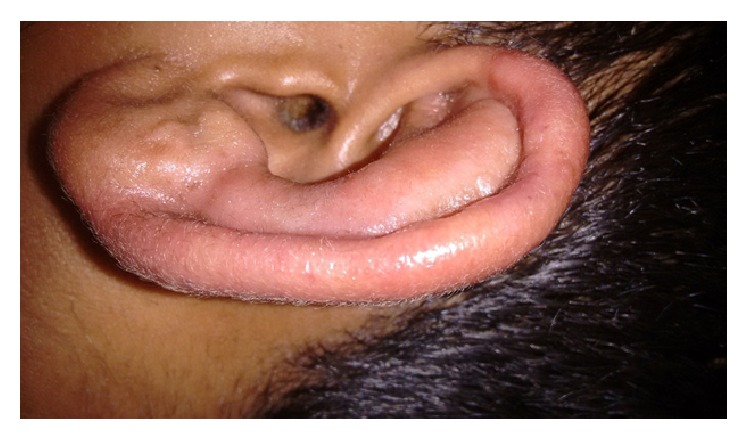
Redness and swelling of cartilaginous upper two-thirds of the external ear.

A 22-year-old male patient from India, with no significant past medical history, presented to us with complaints of fever, pain in small joints of hands with morning stiffness, skin rash over both the elbows and knees, redness and pain in both the external ears, and generalized weakness for one month. His appetite was reduced and he had lost three kilograms of body weight during this period. There was no history of paraesthesia over any body part. He had previously received two courses of broad spectrum antibiotics from his family physician without any relief. He was afebrile at presentation and had normal pulse rate and blood pressure. His general physical examination revealed mild pallor, symmetrical rash over extensor aspects of both the elbows without any loss of sensation (Figure 1) and redness of upper two-thirds of both the external ears with sparing of lobules (Figure 2). There was no palpable nerve thickening. His musculoskeletal examination revealed tenderness over bilateral metacarpophalangeal and interphalangeal joints and his hand squeeze was tender. There was no obvious joint swelling.

His investigations revealed hemoglobin 10.5 g/dl, total leukocyte count 12700/mm^3^, platelet count 179000/mm^3^, and raised inflammatory markers (ESR 112 mm/hour and CRP 11.27 mg/L). His kidney and liver function tests were normal except for reversed albumin/globulin ratio. Urine routine examination showed 10–15 RBCs/hpf and twenty-four hours' urinary protein estimation was 540 mg. Plain radiogram of the chest was normal.

In view of history of auricular chondritis, rash, inflammatory joint pain, constitutional symptoms, and proteinuria his autoimmune antibody profile was done. Complement levels, RF, ANA, and anti-neutrophil cytoplasmic antibodies (ANCA) were negative. His HIV screen was also negative. In view of the typical chondritis of the ear with sparing of noncartilaginous portion, relapsing polychondritis was considered as the possible diagnosis. Granulomatosis with polyangiitis was also kept as a differential but was less likely in view of the absence of sinus and pulmonary involvement and negative ANCA report. Bacterial chondritis was not considered as he had previously received courses of antibiotics without any benefit and had a long history of one-month duration. Trauma and radiation exposure are other rare causes of chondritis which were ruled out by appropriate questioning.

Skin biopsy from the lesions over elbows was done which showed unremarkable epidermis and dermis with perivascular and periadnexal lymphohistiocytic collection and ill formed granuloma. Ziehl–Neelsen stain was positive for acid fast bacilli, and positive Fite-Faraco stain further confirmed the diagnosis of leprosy.

Patient was treated with the combination of clofazimine, dapsone, and rifampicin. He became symptom-free after six months of treatment with complete clearance of skin rash and ear lesions. His joint pain and constitutional symptoms of fever and anorexia have subsided and he has regained his lost weight and feeling of wellbeing. He is still in the maintenance phase of treatment.

## 3. Discussion

Relapsing polychondritis is characterized by episodic inflammation of cartilaginous structures of the body. The most characteristic feature of RPC is painful auricular chondritis of the cartilaginous portion (upper two-thirds) of the pinna with sparing of the lobule [6]. Cartilage involvement in leprosy is well known. Involvement of the external ear and nasal and upper airway cartilage may result in a clinical picture simulating rheumatic diseases like granulomatosis with polyangiitis and RPC [6, 7]. In contrast to RPC, auricular inflammation due to leprosy usually also involves the ear lobules and causes little or no pain [7]. Our patient had painful chondritis with relative sparing of lobule thus mimicking RPC. A variety of skin lesions similar to those of our patient may also be seen in patients of RPC [6].

Immune status of the patient decides the type and number of skin lesions in leprosy.

Lesions in tuberculoid leprosy are usually scanty and symmetrical with smooth borders and have hypoaesthesia [3]. On the other hand lepromatous leprosy seen in patients with poor cell mediated immunity tends to cause multiple, asymmetrical skin lesions with irregular margins [3]. Our patient had atypical skin and ear lesions and did not fit into either the tuberculoid or lepromatous poles of spectrum of the illness. After histopathological confirmation and correlation with the clinical picture the disease was classified as borderline lepromatous leprosy.

Here we highlight the need for a careful physical examination and histopathological confirmation to rule out infectious causes in patients presenting with autoimmune diseases. Peripheral nerves and skin should be carefully examined and tissue diagnosis should be attempted whenever possible.

## Additional Points

Manuscript or any of the figures do not contain any personal information or identification mark by which the identity of the patient may be revealed. Place of research is Asian Institute of Medical Sciences, Faridabad, India. Institutional webpage is http://www.aimsindia.com/about-us/overview/.
